# Production cow-calf responses from perennial forage-based and integrated beef-cropping systems

**DOI:** 10.1093/tas/txac090

**Published:** 2022-06-29

**Authors:** Zac E Carlson, Levi J McPhillips, Galen E Erickson, Mary E Drewnoski, Jim C MacDonald

**Affiliations:** Department of Animal Science, University of Nebraska–Lincoln, Lincoln, NE 68583, USA; Department of Animal Science, University of Nebraska–Lincoln, Lincoln, NE 68583, USA; Department of Animal Science, University of Nebraska–Lincoln, Lincoln, NE 68583, USA; Department of Animal Science, University of Nebraska–Lincoln, Lincoln, NE 68583, USA; Department of Animal Science, University of Nebraska–Lincoln, Lincoln, NE 68583, USA

**Keywords:** annual forage, beef cow, drylot, limit-feeding, partial-confinement, systems

## Abstract

An experiment was conducted to measure production responses of an alternative cow-calf production system integrated into a cropping system without access to perennial forage compared to a traditional cow-calf system utilizing perennial forage. Multiparous, cross-bred beef cows (*n* = 160; average age = 6.2 ± 2.8 yr) were utilized in a randomized complete block experimental design and unstructured treatment design. Upon initiation, cows were blocked by age and stratified by source, assigned randomly to one of two production systems, each with four replicates (*n* = 20 cows/replicate). Once allotted to their treatment groups, cows remained in their experimental units for the duration of the experiment. Treatments were: 1) a traditional system consisting of April to May calving with smooth bromegrass pasture and grazed corn residue as forage resources (TRAD); 2) an alternative system consisting of July to August calving utilizing partial-drylot feeding, summer-planted oats, and corn residue grazing (ALT). There were no differences (*P ≥* 0.27) in calving rates (91.8 vs. 86.7 ± 2.92%), pregnancy rates (89.3 vs. 89.9 ± 2.66%), and weaning rates (87.2 vs. 82.3 ± 3.29%) for TRAD vs. ALT, respectively. However, there was an increase (*P* = 0.04) in the rate of twin offspring in ALT (2.9 vs. 9.4 ± 2.36% for TRAD vs. ALT, respectively). One calf from the set of twins was selected randomly at birth to be removed from the experiment, so the production data are only from single calves. There was no difference (*P* = 0.47) in calf body weight at birth (40 vs. 39 ± 0.7 kg for TRAD vs. ALT, respectively). At weaning, calves in the ALT system were lighter (*P* < 0.01) at the same day of age (184 vs. 229 ± 5.5 kg) compared to TRAD calves. Cows from the ALT system had fewer (*P* < 0.01) kg weaned per cow exposed to bull (150 vs. 199 ± 7.2 kg) compared to TRAD cows. Apart from the twinning rate, no differences in reproductive performance were observed among systems. However, reduced weaning weights and kilogram of weaned calf per cow exposed may negatively impact revenue to the cow-calf enterprise of the ALT system.

## INTRODUCTION

It is estimated that 530,000 hectares of grasslands were converted to corn and soybean production in the northern plains region from 2006 to 2011 (Wright and Wimberly, 2013). Limited pasture availability and record-high corn price resulted in record-high land values in Nebraska in 2014–2015. These elevated land values caused pasture rental rates to rise (Jansen and Stokes, 2020). The reduction in perennial grasslands and increased land values created a need for the use of alternative forages and intensive cow-calf systems. Research has demonstrated that limit-feeding cows in a drylot setting is comparable in cow reproductive performance to traditional pasture cow-calf systems (Loerch, 1996; Anderson et al., 2013, Jenkins et al., 2015; Warner, 2015; Gardine et al., 2019). Loerch (1996) reported that limit-feeding a corn-based diet as an alternative to hay had no negative effects on cow performance, pregnancy rates, and calf weaning weights. Historically, emphasis has been placed on reducing feed costs, primarily by reducing harvested forages and feeds, as that has been considered the greatest variable cost associated with cow-calf systems (Miller et al., 2001). However, utilizing by-products and crop residues is an economical option to maintain cows and cow-calf pairs in drylots (Jenkins et al., 2015; Warner, 2015). Additionally, winter grazing corn residue is an economical alternative to harvested forages or limit-feeding in a drylot for non-lactating cows (Adams et al., 1996; Gardine et al., 2019).

The use of double-cropped annual forages, commonly referred to as cover crops, has increased in popularity over the past decade. From 2012 to 2017, an additional two million ha of cover crops were planted in the United States (SARE/Conservation Technology Information Center, 2020). Cover crops provide several advantages, including soil conservation, weed control, and an alternative forage source for livestock producers (Drewnoski et al., 2018). Grazing late-summer planted cover crops provides economic incentives for livestock owners by providing high-quality forage for cattle and economic incentives for crop producers with grazing rent and no impact on subsequent crop yields (Fae et al., 2009; Anderson et al., 2022). Planting date of cover crops is one of the most significant factors determining forage production of the late-summer planted cover crop with earlier planting resulting in greater yields (Koch et al., 2002). Energy and protein content of late-summer planted cool-season winter sensitive annuals such as oats remains elevated in early winter (Lenz et al., 2019), whereas corn and soybeans are the predominant crops planted in Nebraska and other Midwest states. Production and harvest of wheat, seed corn, and corn silage can provide an opportunity for producers to capitalize on late-summer planted cover crops as a double-crop annual forage. In Nebraska, 41% of cover crops were planted after wheat, seed corn, and corn silage (Drewnoski et al., 2015).

We hypothesized that an alternative cow-calf production system could be developed for producers without access to perennial forages by utilizing confined feeding, double-cropped annual forages, and crop residues. Our objective was to compare a traditional cow-calf system utilizing perennial forage and corn residue grazing to an alternative cow-calf system utilizing confined feeding, late-summer planted oats grazing, and corn residue grazing on cow reproduction and calf performance.

## MATERIALS AND METHODS

### Use of Animal Subjects and Experiment Site

**Figure 1.**
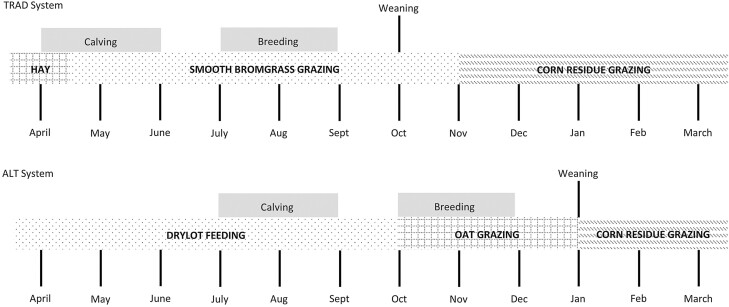
Annual timeline of feed resources and cow production cycles for two cow-calf production systems. Treatments consisted of a traditional, spring-calving cow-calf system utilizing smooth bromegrass pasture in spring, summer, and fall, and winter corn residue grazing (TRAD) and an alternative fall-calving cow-calf system utilizing partial-drylot, fall grazing of a late-summer planted oat cover crop and corn residue grazing (ALT).

All facilities and management procedures used in this experiment were approved by the University of Nebraska—Lincoln Institutional Animal Care and Use Committee (IACUC # 1491). This experiment was conducted over two production cycles (calving to weaning) at the Eastern Nebraska Research and Extension Center (ENREC) near Mead, Nebraska. Multiparous, cross-bred beef cows (*n* = 160; average age = 6.2 ± 2.8 yr) were utilized in a randomized complete block design with two treatments. Cows originated from two separate herds at ENREC and were managed in spring-calving, forage-based systems. Cows were blocked (*n* = 4) by cow age and then stratified by origin source (two sources) within block and assigned randomly within strata to one of two production system treatments with four replicates. Once allocated to treatment and replicate, cows remained in their experimental unit for the duration of the experiment or until removed for failure to wean a calf. Treatments included: 1) a traditional system with April to May calving, utilizing smooth bromegrass (*Bromus inermis*)–based pastures and corn residue grazing (TRAD), or 2) an alternative system with July to August calving, utilizing confined feeding in the spring and summer, fall grazing of late summer-planted oats (*Avena sativa*, var. *goliath*), and corn residue grazing in the winter (ALT). An annual timeline of feed resource use and cow production cycle for the TRAD and ALT systems is shown in Figure 1.

Prior to the experiment, all cows were spring-calving. Therefore, at the time cows were assigned to and started treatments (July of 2017), cows in the ALT treatment had to be switched from spring-calving to summer-calving and breeding was delayed from July to October. Thus, during the first breeding season, cows in the ALT treatment had just weaned a calf and unlike the TRAD, were not lactating. Therefore, data from the 2017 breeding season for both treatments were not included in the results. The pregnancy rates from the 2017 breeding season were 98.8% and 95.0% for the ALT and TRAD cows, respectively. The calving data from the calves conceived during the 2017 breeding were the start of the experimental data collection.

### Cow-Calf Production Systems

#### Alternative system.

The ALT system was designed to be a summer-calving system with calving occurring in the drylot during the time of year with historically less precipitation. An objective of the ALT treatment design was to use late summer-planted oats to meet the nutrient requirements of the cows during lactation and breeding in the fall. The ALT system utilized spring and summer limit-feeding in a drylot, fall grazing of forage oats, and winter corn residue grazing (Figure 1). During the drylot period, cows in the ALT treatment were housed in open feedlot pens with approximately 76 cm of linear bunk space and 79 m^2^ of pen space per cow. Cows were limit-fed to meet nutritional requirements based on physiological stages during gestation and lactation periods (NASEM, 2016). Table 1 shows the diet composition, Table 2 shows the composition of the supplement used in the diets, and Table 3 shows the amount of diet fed. Cows were fed once daily between 0900 and 1200 h with ad libitum access to fresh water. In both cycles, the limit-fed diet was formulated to provide 200 mg/cow daily of monensin (Rumensin 90; Elanco Animal Health, Greenfield, IN; Table 2). Diets were mixed and delivered using a truck-mounted feed mixer and delivery unit with scale measurements to the nearest 0.45 kg (Roto-Mix model 420, Roto-Mix, Dodge City, KS). All scales used for the experiment were calibrated twice annually.

**Table 1.** Composition (DM basis) of limit-fed diet provided during the drylot period (March to October) of the alternative cow-calf system^1^

Ingredient, %	Cycle 1	Cycle 2
MDGS^2^	55.00	54.45
Low quality forage^3^	40.00	40.55
Supplement	5.00	5.00
Nutrient composition, %		
Organic matter	90.76	90.79
Crude protein	19.79	20.93
Neutral detergent fiber	53.81	48.84
Acid detergent fiber	35.07	32.37
Ether extract	5.22	4.86

Treatment = alternative cow-calf system (ALT) calving in July to September and utilizing drylot, fall forage oat grazing, and corn residue grazing.

Modified wet distillers grains plus solubles.

Low-quality forage for cycle one was wheat straw, and cycle two was wheat straw for 73 d, oat hay for 137 d, and ground corn residue for 14 d.

**Table 2.** Ingredient composition (DM basis) of the supplement used in the limit-fed diet during the drylot period of the alternative cow-calf system^1^

Ingredient, %	Cycle 1		Cycle 2	
	Gestation^2^	Lactation^3^	Gestation^2^	Lactation^3^
Fine ground corn	2.47	2.44	2.49	2.45
Beef trace mineral and salt premix^4^	—	—	1.79	1.79
Limestone	1.98	1.98	0.57	0.57
Salt	0.30	0.30	—	—
Tallow	0.125	0.125	0.125	0.125
Beef trace minerals^5^	0.10	0.10	—	—
Insect growth regulator^6^	—	0.0275	—	0.0275
Vitamin A-D-E^7^	0.015	0.02	0.015	0.02
Monensin^8^	0.0138	0.0138	0.0158	0.0158

Alternative cow-calf system was July to September calving with feeding in drylot from mid-March to late October, grazing late summer planted oats from late October to early January, and corn residue grazing from early January to mid-March.

Fed from mid-March to mid-July.

Fed from mid-July to late-October.

Premix contained 21.5% salt, 30.5% Ca, 0.22% Zn, 0.22% Mn, 0.11% Cu, 0.0005% I, 0.0002% Co, and 0.0001% Se.

Premix contained 10% Mg, 6% Zn, 4.5% Fe, 2% Mn, 0.5% Cu, 0.3% I, and 0.05% Co.

JustiFLY feedthrough, Champion Farmoquimico LTDA, Anapolis, Goias, Brazil. Formulated to provide 5 g/kg.

Premix contained 1,500 IU of vitamin A, 3,000 IU of vitamin D, and 3.7 IU of vitamin E per gram.

Rumensin 90, Elanco Animal Health, Indianapolis, IN. Formulated to provide 27.5 mg/kg.

**Table 3.** Dry matter intake of the limit fed diet during drylot period of the alternative cow-calf system^1^

	Cycle 1	Cycle 2
DOF	222	224
DMI^2^, kg/d	7.12	7.53
Gestation period DMI^3^, kg/d	6.44	6.94
Lactation period DMI^4^, kg/d	7.94	8.48

Alternative cow-calf system was July to September calving with feeding in drylot from mid-March to late October, grazing late summer planted oats from late October to early January, and corn residue grazing from early January to mid-March.

Average intake for the entire confinement period.

Diet fed from 16 March to 18 July 2018 (cycle one); 14 March to 17 July 2019 (cycle two).

Diet fed from 19 July to 22 October 2018 (cycle one); 18 July to 22 October 2019 (cycle two).

The ALT cows entered the drylot on 14 March 2018 and 16 March 2019 in cycles one and two, respectively. The ALT cows calved in the drylot from 16 July to 12 September 2018 and 20 July to 28 September 2019 in cycles one and two, respectively. Cow-calf pairs remained in the drylot until 23 October of both cycles for a total of 222 and 224 d in cycles one and two, respectively.

Breeding occurred from 18 October to 17 December 2018 (61 d; cycle one) and 18 October to 17 December 2019 (61 d; cycle two). The first 5 d of breeding occurred in the drylot, and then cows and bulls were relocated to late summer-planted oats fields for the remainder of the breeding season on 17 December in both cycles. The oats had been no-till drilled, following wheat harvest, at a seeding rate of 108 kg/ha on 17 August 2018 (cycle one) and 112 kg/ha on 20 August 2019 (cycle two). Nitrogen, in the form of urea, was surface applied to all oat fields at a rate of 49 kg/ha on 13 August (cycle one) and 37 kg/ha on 14 August (cycle two). In cycle one, oat fields had an infestation of fall armyworm (*Spodoptera frugiperda*) and required an insecticide application.

Stocking rates for the oat fields were approximately 1.19 ha/cow (1.15 to 1.27 ha; cycle one) and 1.16 ha/cow (0.98 to 1.41 ha; cycle two). Each replicate of cow-calf pairs had full access to their assigned oats field. Cow-calf pairs were removed from oats fields when it was visually estimated that forage height was 5.1 cm. In the event forage height reached 5.1 cm prior to weaning, cow-calf pairs returned to the feedlot and were provided the same limit-fed diet at the same intake amount they received prior to grazing oats. The ALT cows grazed oats from 23 October 2018 to 13 January 2019 and 23 October 2019 to approximately 8 January 2020 in cycles one and two, respectively. On the first day of oat grazing, calves in the ALT system were 83 and 74 d of age (cycles one and two, respectively). During cycle two, cow-calf pairs from replicates one and three were relocated to the drylot and limit-fed for 34 and 23 additional d until calves were weaned. The average d of oats grazing was 82 d in cycle one and 77 d in cycle two (range of 58 to 92 d in cycle two).

Calves from the ALT treatment were weaned on 15 January 2019 and 25 January 2020, cycles one and two, respectively. After weaning, cows were moved to corn residue fields on 20 January 2019 and 25 January 2020, cycles one and two, respectively. Stocking rates on corn residue fields were 1.17 and 1.21 ha/cow for cycles one and two, respectively, and grazing days were 49 and 48 d for cycles one and two, respectively. Corn residue grazing ceased on 14 March 2018 and 16 March 2019 in cycles one and two, respectively, and cows were relocated to the drylot to begin the limit-feeding portion of the system.

In the event of snow accumulation sufficient to prevent adequate grazing of corn residue, cows were fed the limit-fed diet. When grazing oats, cows were provided access to high magnesium free-choice mineral. When grazing corn residue, cows were provided with a free-choice mineral. Intake of free-choice minerals was controlled by the addition of salt for a targeted intake of 56 to 114 g/cow, daily.

#### Traditional system.

The TRAD system was designed to be a late-spring calving system with calving occurring on smooth bromegrass pasture. The TRAD system utilized early-spring hay feeding, late-spring, summer and fall grazing on smooth bromegrass pasture (*Bromus inermis*), and winter corn residue grazing (Figure 1).

The calving season was from 10 April to 16 June 2018 and 5 April to 6 June 2019 in cycles one and two, respectively. Cows were comingled prior to calving and fed ground grass hay provided at 13.5 kg for 31 d (cycle one) and 9.3 kg for approximately 81 d (cycle two) on dormant smooth bromegrass pastures until forage was ready for grazing. The TRAD cows began grazing smooth bromegrass pastures on 7 May 2018 and 2 May 2019 in cycles one and two, respectively. Cows were stocked at 1.21 ha/cow in both cycles and grazed for approximately 186 and 197 d for cycles one and two, respectively. Pastures consisted of an average of 24.84 ha and was divided between two and four paddocks. Paddocks were rotationally grazed. Cows were rotated paddocks when the pasture was visually appraised for adequate biomass removed. Nitrogen, in the form of urea, was surface applied to all pastures at a rate of 90 kg/ha in April each year. Breeding occurred from 6 July to 4 September 2018 (61 d; cycle one) and 5 July to 3 September 2019 (61 d; cycle two).

Calves from the TRAD treatment were weaned on 16 October 2018 and 11 October 2019 in cycles one and two, respectively. After weaning, cows returned to smooth bromegrass pastures until the corn was harvested and corn residue fields were ready to graze. On 15 and 8 November in cycles one and two, respectively, cows in the TRAD treatment started grazing corn residue. Corn residue fields were stocked at 1.69 and 1.43 ha/cow in cycles one and two, respectively, and grazed for 119 and 123 d in cycles one and two, respectively. On approximately 17 March 2018 and 12 March 2019 in cycles one and two, respectively, cows were relocated from corn residue fields to dormant perennial pastures in preparation for calving. In the event of snow accumulation sufficient to prevent adequate grazing of corn residue, cows were fed hay. Throughout the year, cows were provided access to free-choice mineral. During the spring, a high-magnesium free-choice mineral was provided. Intake of free-choice mineral was controlled by the addition of salt for a targeted intake of 56 to 114 g/cow, daily.

### Breeding, Health, and Weaning Management

Cows were culled from both systems if they failed to wean a calf and were replaced with cows that had been sourced from one of the original cow herds and were bred within the system they were entering. To accomplish this, an additional replicate was maintained for each system so that replacement cows entered the experiment after being maintained in that respective system for approximately 1 yr. The number of cows replaced during the first cycle were five and eight for ALT and TRAD, respectively, and during the second cycle were twelve and nine for ALT and TRAD, respectively.

Approximately 1 mo before breeding, cows in both treatments were vaccinated against infectious bovine rhinotracheitis caused by infectious bovine rhinotracheitis (IBR) virus, bovine viral diarrhea caused by bovine viral diarrhea (BVD) Type one and two virus, disease caused by parainfluenza3 (PI3) virus and bovine respiratory syncytial virus (BRSV), campylobacteriosis (vibriosis) caused by *Campylobacter fetus*; and leptospirosis caused by *Leptospira canicola, L. grippotyphosa, L. hardjo, L. icterohaemorrhagiae,* and *L. Pomona* (Bovi-Shield Gold FP 5 VL5, Zoetis, Parsippany, NJ).

Cows from both systems were treated annually in April for with 1% doramectin (Dectomax, Zoetis) for control of internal and external parasites. Approximately 1 mo before calving, cows were vaccinated against bovine rotavirus (serotypes G6 and G10), bovine coronavirus, enterotoxigenic strains of *Escherichia coli* having the K99 pili adhearance, and *Clostridium perfringens* type C (Scourguard 4KC, Zoetis).

Cows from both systems were exposed to the same set of Simmental × Angus bulls that had passed an annual breeding soundness exam, performance by a licensed veterinarian, 30 d prior to each breeding season. Two bulls were allocated to each replicate of cows to prevent reproductive failure due to inadequate bull performance (in case of injury or lack of libido during the breeding season). Therefore, the bull:cow ratio was 1:10. The breeding season for both treatments was 63 and 61 d for cycles one and two, respectively. The two bulls with the highest calving ease expected progeny differences were allocated to the youngest replicate of cows for each treatment for both cycles. The remaining bulls were assigned randomly to one replicate and re-randomized each cycle. All cows were given 5 mL of prostaglandin F_2α_ (5 mg/mL dinoprost tromethamine, Lutalyse, Zoetis Animal Health, Parsippany, NJ) following 5 d of bull exposure (Whittier et al., 1991). Pregnancy was diagnosed via BioPRYN (BioTracking, LLC, Moscow, ID) pregnancy detection blood test at 31 d (TRAD; cycle one), 29 d (ALT; cycle one), 52 d (TRAD; cycle two), and 50 d (ALT; cycle two) after bulls were removed.

Calves were vaccinated upon birth against respiratory disease caused by bovine respiratory syncytial virus (BRSV), infections bovine rhinotracheitis (IBR) virus, and parainfluenza (PI_3_) virus (Inforce 3; Zoetis), against blackleg caused by *Clostridium chauvoei,* malignant edema caused by *Clostridium septicum,* black disease caused by *Clostridium novyi,* gas-gangrene caused by *Clostridium sordellii*, enterotoxemia and enteritis caused by *Clostridium perfringens* types B, C, and D (Ultrabac 7; Zoetis), treated against omphalitis and omphalophlebitis by exposing the navel to 7% tincture of iodine solution (Vetericyn Super7+ Navel Dip; Vetericyn Animal Wellness, Rialto, CA), and received a panel tag in the right ear with an individual identification number. At birth, a manual, portable scale (Salter-Brecknell Model 235, Avery-Weigh Tronix LLC, Fairmont, MN) was used to capture birth BW. If a cow gave birth to twins, one calf was selected randomly and removed from the experiment at birth.

Calves from both systems were weaned using a fence-line weaning strategy. All calves from the four replicates within a treatment were comingled in a pen. Cows from two replicates within treatment were held in an adjacent pasture to provide visual and auditory stimulation for the weaned calves. Calves were fence-line weaned for three d and limit-fed grass hay at 2.0% of BW before being transported to the ruminant nutrition feedlot at ENREC. At the feedlot, calves were limit fed (at approximately 2.0% BW) a diet consisting of 50.0% Sweet Bran (Cargill Corn Milling, Blair, NE) and 50.0% alfalfa hay (DM basis) to minimize variation in gastrointestinal fill (Stock et al., 1983; Watson et al., 2013) for 5 d followed by two consecutive days of BW measurements (Silencer squeeze chute, Moly Mfg. Inc., Lorraine, KS).

### Animal Performance and Health Calculations

Cow body condition scores (BCS) were assigned by two trained observers using a 9-point scale (1 = emaciated, 9 = obese) at the beginning of breeding and at weaning. Cow replacement rate was determined by the number of cows per group that were removed from the experiment due to failure to wean a calf divided by the total number of cows in that respective group. Mortality percentage was calculated by total number of animals that died in a group divided by the total number of animals in that respective group. Calves that died at birth or within the first 24 h of life were not included in mortality calculations, those deaths were accounted for in the calving percent calculations. Percentage of animals removed from experiment, excluding dead, was determined by dividing the number of animals removed due to injury or chronic illness per group by the total number of animals from that respective group. Morbidity percentage was calculated as the number of animals in a group that were treated at least once, divided by the total number of animals in that respective group. Mortality and morbidity for cows and calves are reported separately.

### Feed and Forage Sample Collection and Analysis

Feed ingredient samples of the drylot diet were collected weekly, weighed, and then dried in a 60 °C forced-air oven to determine DM content (AOAC, 1999; method 934.01). Dried feed samples were ground through a 1-mm screen with a Wiley mill (Model 4 Thomas Scientific, Swedesboro, NJ) and composited by month. Ash and OM were measured by placing crucibles containing 0.5 g of each feed ingredient sample in a muffle furnace for 6 h at 600 °C (AOAC, 1999; method 945.05). Neutral and acid detergent fiber analyses were conducted using the procedures by ANKOM Technologies (2017). Crude protein (CP) was also analyzed using a combustion-type N analyzer (FlashSmart N/Protein Analyzer CE Elantech, Inc., Lakewood, NJ).

In cycles one and two, diet samples of the bromegrass pasture were collected during the grazing season from one replicate. On the day cow-calf pairs were rotated into a new paddock, two ruminally cannulated steers were used to sample pastures prior to grazing. Steers were ruminally evacuated at 0800 h on each sampling day. Steers were given 30 min to graze each paddock or pasture, and then brought back to the handling facility where masticate samples were collected and immediately put on ice and rumen contents returned to the rumen. In cycle two, smooth bromegrass was sampled for biomass production throughout the grazing season as cows rotationally grazed. Prior to rotation, total biomass was measured from the current pasture (post-grazed) and the pasture rotated to next (pre-grazed) by randomly selecting four (0.91 × 0.91 m^2^) areas within one replication’s allocated pasture.

Initial oat biomass was sampled on 29 October 2018 (cycle one) and 21 October 2019 (cycle two). Biomass samples were collected before (October) and after (January) grazing for each replication in cycle one and collected before (October), during (November), and after (January) grazing for each replication in cycle two. Total biomass was measured by randomly selecting (0.91 × 0.91 m^2^) four areas within each replication’s field. Forage was clipped at ground level, bagged, and dried for 48 h in a 60 °C forced-air oven to determine pre-and post-grazing biomass. Oat samples for nutrient analysis were clipped at ground level and collected in October, prior to grazing, and in late-January, post-grazing. Each sample was collected at random within each replication’s field and put into separate bags for a total of four samples for both pre-and post-grazing.

All forage samples were frozen at −4 °C until being lypholized at −50 °C (Virtis Freezemobile 25ES, Life Scientific Inc., St. Louis, MO) and ground through a 1-mm screen using a Wiley mill (Model 4; Thomas Scientific). Freeze-dried samples were analyzed for 100 °C corrected dry matter. In vitro organic matter digestibility was determined for 48 h using the method described by Tilley and Terry (1963). Samples were prepared in triplicate and the procedure was repeated to provide two replications per sample. The IVOMD method by Tilley and Terry (1963) was modified with the addition of urea to the McDougall’s buffer (McDougall, 1948) at a rate of 1 g urea/L of buffer solution to ensure adequate N was available for microbes in the rumen fluid (Weiss, 1994). Rumen fluid was collected from two ruminally cannulated donor steers provided a mixed diet of 70% bromegrass hay and 30% DGS. After incubation, samples were filtered using a filter paper with particle filtration of 22 µm (Whatman Grade 541; Cytiva, Marlborough, MA) and dried at 100 °C to determine DM disappearance. Samples were then placed in crucibles and heated in a muffle furnace for 6 h at 600 °C to determine OM disappearance (AOAC, 1999; method 4.1.10). Blanks were included in the in vitro run to adjust for any feed particles that might have come from the inoculum. Five grass hay standards with known in vivo (total tract) digestibility (51%–60% range) were used to adjust IVOMD values (Stalker et al., 2013). These adjustment values resulted in −0.00581 percentage units added to IVOMD.

Crude protein (CP) was analyzed in the forage samples using a combustion-type N analyzer (FlashSmart N/Protein Analyzer CE Elantech, Inc., Lakewood, NJ).

### Statistical Analysis

Cow performance and calf growth performance data were analyzed using the GLIMMIX procedures of SAS (SAS Institute Inc., Cary, NC), where replicate was considered the experimental unit (*n* = 8 replicates/treatment). Cows were blocked by cow age and stratified by the original source (two sources). The model included treatment and block as a fixed effect and cycle as a random effect. The proportion of heifers and twins were tested as covariates but were not significant (*P* > 0.11) and subsequently removed from the model.

Reproduction and morbidity data were analyzed using the GLIMMIX procedure of SAS 9.4 (SAS Institute Inc.) with a binomial model with replicate as the experimental unit and fixed effects of treatment and block. Cycle was included as a random effect. The model for reproductive data specified a solutions function for the binomial response, with the number of cows exposed to bulls per replicate as the denominator. The model for morbidity data specified a solutions function for the binomial response, with the total number of calves per replication serving as the denominator. Body condition scores were analyzed using the GLIMMIX procedure of SAS 9.4 (SAS Institute Inc.) with a multinomial model with fixed effects of treatment and block. Cycle was included as a random effect. The model specified a solutions function for the multinomial response, with the number of animals scored identified in the denominator.

Forage quality, estimated by IVOMD and CP, for pasture diet samples and fall oat clipped samples, and biomass production for pasture were analyzed using PROC MIXED procedure of SAS (SAS Institute Inc., Cary, NC). Regression was used to determine linear or quadratic effects of IVOMD and CP over time during the grazing season. Cycle was considered a random effect.

## RESULTS AND DISCUSSION

### Climate

High and low monthly temperature and monthly rainfall are presented in Table 4. In 2018, in cycle one of the experiment, the climate in Lincoln, NE consisted of temperatures ranging from a low of −28.3 °C in January to a high of 38.3 °C in June. Total precipitation from January to December was 90.2 cm, with a monthly high of 22.4 cm in June and a low of 1.0 in January (NWS, 2021). In 2019, in cycle two of the experiment, the climate in Lincoln, NE consisted of temperatures ranging from a low of −22.8 °C in March to a high of 37.2 °C in June and again in July. Total precipitation from January to December was 92.4 cm, with a monthly high of 18.5 cm in May and a low of 1.8 cm in January (NWS, 2021).

**Table 4.** Monthly temperature (°C) and precipitation (cm) over 2 yr for two different cow-calf systems^1^

Item	Temperature (°C)				Precipitation (cm)		System^2^		
	Low	High	Low	High					
	2018	2018	2019	2019	2018	2019	30-yr^3^	TRAD	ALT
January	−28.3	13.3	−22.8	16.1	1.04	1.75	1.85	—	Weaning
February	−21.1	18.3	−21.7	14.4	1.88	4.04	2.26	—	—
March	−11.1	22.8	−22.8	24.4	6.73	6.73	3.94	—	—
April	−12.2	27.8	−4.4	30.6	1.70	2.92	6.83	Calving	—
May	6.1	37.8	1.7	34.4	5.66	18.52	12.47	Calving	—
June	11.1	38.3	7.8	37.2	22.43	11.13	11.38	Calving	—
July	13.9	36.1	7.8	37.2	3.43	11.13	8.26	Breeding	Calving
August	10.0	35.6	10.6	35.0	11.05	7.09	8.43	Breeding	Calving
September	5.0	35.6	8.9	34.4	18.11	8.64	7.37	Breeding	Calving
October	−2.8	34.4	−7.8	26.1	6.88	11.91	5.44	Weaning	Breeding
November	−14.4	17.2	−16.7	23.3	3.02	2.01	3.30	—	Breeding
December	−15.6	13.3	−12.8	15.6	8.23	6.53	3.00	—	Breeding

All data were acquired from https://www.weather.gov/oax/monthly_climate_records.

Treatments = alternative cow-calf system (ALT) calving in July to September and utilizing drylot, fall forage oat grazing, and corn residue grazing; traditional cow-calf system (TRAD) calving in April to June and utilizing perennial forage and corn residue grazing.

30-yr historical precipitation from 1991 to 2020 from NOAA (2020).

### Forage Quality and Quantity

**Figure 2.**
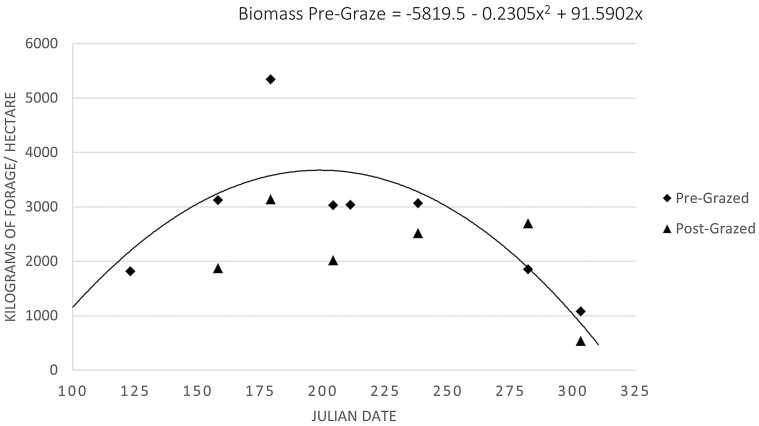
Smooth bromegrass pre-grazed biomass production quadratically increased (*P* = 0.05; SE = 0.091) over the grazing season in cycle two for the traditional cow-calf system. There were no differences (*P* = 0.39; SE = 6.340) for post-grazed biomass measurements. The grazing season for the traditional cow-calf system was Julian days 122 (2 May 2019) to 312 (8 November 2019) for cycle two.

**Figure 3.**
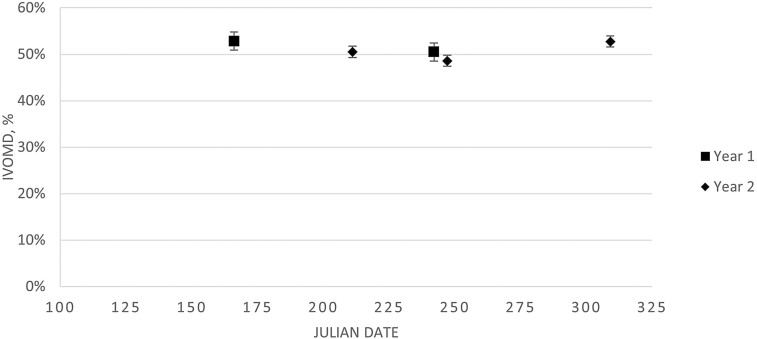
Smooth bromegrass, collected via diet sample, in vitro organic matter digestibility (IVOMD) was not different (*P* = 0.94; SE = 0.0195) over the grazing season for the traditional cow-calf system. The grazing season for the traditional cow-calf system was Julian days 127 (7 May 2018) to 319 (15 November 2018) and 122 (2 May 2019) to 312 (8 November 2019) for cycles one and two, respectively.

**Figure 4.**
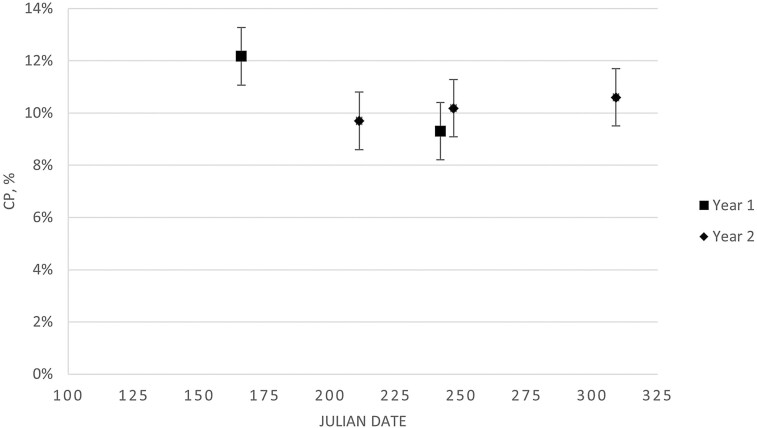
Smooth bromegrass, collected via diet sample, crude protein (CP) was not different (*P* = 0.19; SE = 0.011) over the grazing season for the traditional cow-calf system. The grazing season for the traditional cow-calf system was Julian days 127 (7 May 2018) to 319 (15 November 2018) and 122 (2 May 2019) to 312 (8 November 2019) for cycles one and two, respectively.

Smooth bromegrass biomass availability for cycle two is presented in Figure 2. Over time, smooth bromegrass availability quadratically increased (*P* = 0.05) with July having the greatest biomass. This agrees with previous biomass data from smooth bromegrass pastures at this location (Greenquist et al., 2009). There were no differences (*P* = 0.94) in smooth bromegrass IVOMD and CP over the grazing season for cycles one and two (Figures 3 and 4, respectively). An average IVOMD of approximately 50% and CP of approximately 10% suggest that the TRAD cows had access to a moderate quality diet throughout the summer grazing season. The effects of forage quality across the grazing season of smooth bromegrass pastures, represented as IVOMD and CP, from the current experiment differs from previous research on smooth bromegrass pastures at this location. Greenquist et al. (2009) reported a quadratic effect for in vitro dry matter digestibility (IVDMD) of smooth bromegrass with the highest digestibility in late April (71.6%) and lowest digestibility in August (54.6%) with an increase at the end of the grazing season in September (57.1%). Conversely, Watson et al. (2012) reported a linear decrease in IVDMD of smooth bromegrass from 65.6% to 51.4% from May to September, respectively. Greenquist et al. (2009) observed a cubic effect for CP, with late April having the highest CP (18.8%), decreasing to July (13.3%), and then increasing to September (17.5%). Watson et al. (2012) observed a linear decrease in CP content from May to September (18.4% to 14.5%, respectively). It should be noted that the previous work by Greenquist et al. (2009) and Watson et al. (2012) was performed on the same paddocks and managed with more frequent rotations among paddocks than the rotation cycle used in the current experiment. Likewise, smooth bromegrass samples evaluated from the current experiment were sampled from June to November, which was closer to the actual grazing season for the TRAD system compared to the sampling timepoints reported by Greenquist et al. (2009) and Watson et al. (2012).

**Figure 5.**
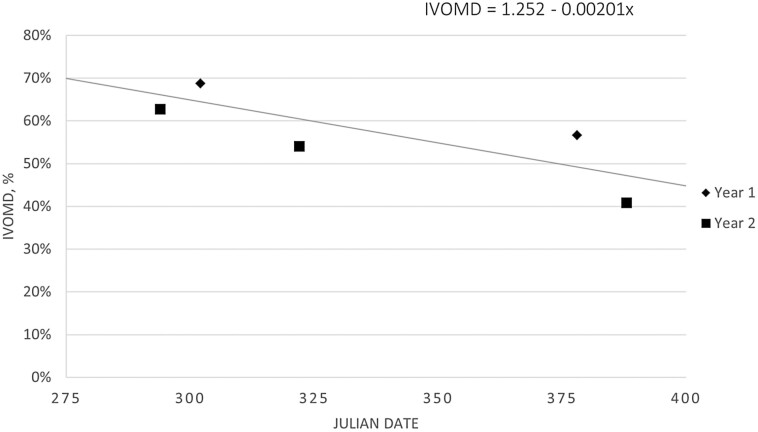
Late-summer planted oat, clipped at ground level, in vitro organic matter digestibility (IVOMD) linearly decreased (*P* = 0.02; SE = 0.0272) over the grazing season for the alternative cow-calf system. The oats grazing season for the alternative cow-calf system was Julian days 296 (23 October 2018) to 378 (13 January 2019) and 296 (23 October 2019) to 373 (8 January 2020) for cycles one and two, respectively.

**Figure 6.**
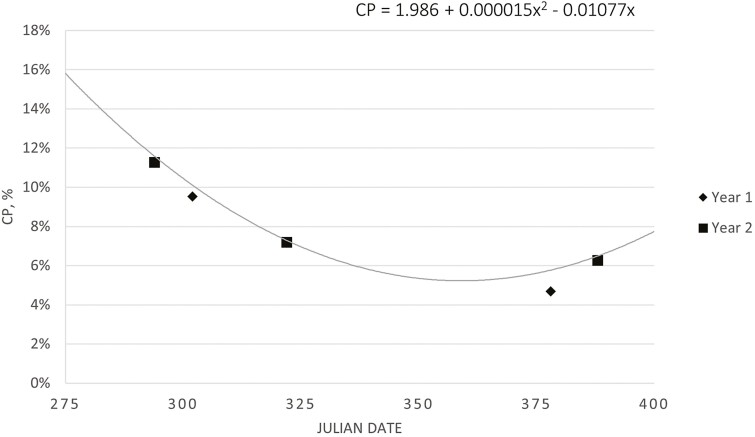
Late-summer planted oats, collected via clipped sample, crude protein quadratically decreased (*P* = 0.04; SE = 0.0003) over the grazing season for the alternative cow-calf system. The oats grazing season for the alternative cow-calf system was Julian days 296 (23 October 2018) to 378 (13 January 2019) and 296 (23 October 2019) to 373 (8 January 2020) for cycles one and two, respectively.

Oat forage biomass was 3,213 and 2,588 kg/ha for cycles one and two, respectively. Oat IVOMD decreased linearly (*P* = 0.02) over the grazing season from approximately 65% to 48% (Figure 5). This agrees with previous research (Lenz et al., 2019), which reported decreased IVOMD in January (67.4%) compared to October (79.0%) for oats planted in late August and early September. In the current experiment, crude protein for mid-August planted oats decreased quadratically (*P* = 0.04) over the grazing season from approximately 11% to 6% (Figure 6). Lenz et al. (2019) did not observe a change in CP of oats, ranging of 13.8% to 17.9%, for oats planted in late August and early September. The higher IVOMD and CP values reported by Lenz et al. (2019) may be due to later planting dates compared to the current experiment.

### Cow-Calf Performance

Cow reproduction and production results are presented in Table 5. Cow age had a tendency (*P* = 0.06) to be different with ALT cows 0.3 yr older than TRAD. This tendency is likely because replacement cows were not always the same age as those removed from the experiment. Cow morbidity and replacement rates did not differ among systems (*P* ≥ 0.78). There were no significant differences (*P* ≥ 0.27) for pregnancy, calving, or weaning rates. These results agree with previous research reporting no difference in pregnancy rates for confined cow-calf systems compared to forage-based cow-calf systems (Anderson et al., 2013; Neira, 2019).

**Table 5.** Comparison of an extensive, spring-calving cow-calf system to a partial-intensive, fall-calving cow-calf system on cow performance

	Treatment^1^		SEM	*P*-Value
	ALT	TRAD		
Groups, *n*	8	8	--	--
Age, yr	6.3	6.0	0.49	0.06
Calving rate^2^, %	89.7	91.2	2.92	0.71
Twin rate^3^, %	9.4	2.9	2.36	0.04
Pregnancy rate^4^, %	89.3	89.9	2.66	0.88
Wean rate, %	82.3	87.2	3.29	0.27
Cow morbidity^5^, %	18.9	17.6	3.24	0.78
Cow mortality, %	0.6	0.6	—	—
Replacement rate^6^, %	9.6	9.9	2.89	0.93

Treatments = alternative cow-calf system (ALT) calving in July to September and utilizing drylot, fall forage oat grazing, and corn residue grazing; traditional cow-calf system (TRAD) calving in April to June and utilizing perennial forage and corn residue grazing.

Calving data are from July to September 2018 and 2019 for the ALT system and April to June 2018 and 2019 for TRAD.

One calf from each set of twins was selected randomly and removed from the experiment.

Breeding data are from October to December 2018 and 2019 for the ALT system and July to September 2018 and 2019 for the TRAD system.

Number of cows treated for morbidity at least once.

Percentage of cows removed from the herd due to failure to breed or wean a calf.

Several studies have found that limit-feeding high-energy diets comprised of corn or ethanol co-products to cows during late gestation or early lactation do not affect reproductive performance (Loerch, 1996; Tjardes et al., 1998; Schoonmaker et al., 2003; Shike et al., 2009; Warner, 2015). Most of these studies ended the limit-feeding phase at the beginning of the breeding season. In the current experiment, the limit-feeding phase ended at the beginning of the breeding season but resumed when cows were in mid-gestation and continued through early lactation. Schoonmaker et al. (2003) limit-fed cows a diet of corn, soybean meal, and orchardgrass hay prior to a 45 d breeding season, but breeding occurred on pasture. The authors reported no differences in pregnancy rates for cows previously limit-fed or fed ad libitum grass hay or pasture (86.1%, 91.4%, and 96.0%, respectively). These findings support the data in the current experiment, indicating that limit-feeding programs prior to breeding have acceptable breeding performance provided the diet meets the cow’s nutrient requirements.

In the current experiment, there were no differences (*P* = 0.76) in the proportion of calves born that were heifers among treatments (49.7% vs. 51.5% for TRAD and ALT, respectively; data not shown). However, cows from the ALT system had a greater probability of producing twin offspring (*P* = 0.04) than TRAD cows (Table 5). This response was unexpected. Cows in the ALT system had more than three times the number twins than TRAD cows (9.4% vs. 2.9%, respectively). The incidence of twins can be linked to physiologic and genetic components (Fricke, 2001). Given our allocation method, it is unlikely that the twinning response to the ALT treatment can be explained by the genetic differences of cows enrolled in the treatment. Cows utilized in the experiment originated from two herds within the University of Nebraska system and were stratified by source to treatments. Previous information on the herds suggests a normal twinning rate, similar to the observed rate reported for the TRAD system (2.9%). Additionally, if the cause were solely linked to genetic differences, it would be expected that the same cows would have twins in multiple years. In the current experiment, cows that twinned in cycle one were not the same as those that twinned in cycle two.

Twinning and ovulation are strongly associated with cattle (Morris et al., 1986). Echternkamp et al. (1990) reported greater ovulation rates in beef cows in the fall compared to spring. Additionally, it has been suggested that feed intake may increase the hepatic metabolism of ovarian steroids in lactating dairy cows, a response also observed in ewes (McEvoy et al., 1995; Parr et al., 1993). In the current experiment, during the first five d of breeding, cows remained on the limit-fed diet and then grazed oats fields for the remainder of breeding. Although ovulation rates in the current experiment are unknown, the combination of breeding in the fall (October to December) and diet change at the beginning of the breeding season may contribute to the increase in twin offspring for the ALT system.

**Figure 7.**
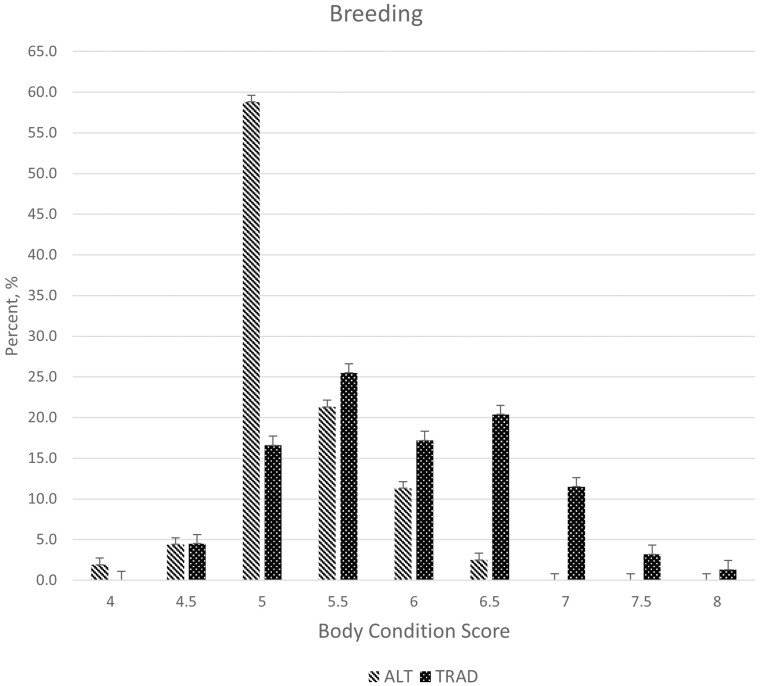
Body condition score at the beginning of the breeding season for two cow-calf production systems. Treatments consisted of an alternative fall-calving cow-calf system utilizing partial-drylot, cover crop, and corn residue grazing (ALT) and a traditional, spring-calving cow-calf system utilizing perennial forages and corn residue grazing (TRAD). Body condition distribution was significantly (*P* < 0.01; SE = 0.82) different among the treatments. There was a shift in the ALT body condition distribution with a greater percentage near a body condition score of 5 compared to the TRAD treatment.

**Figure 8.**
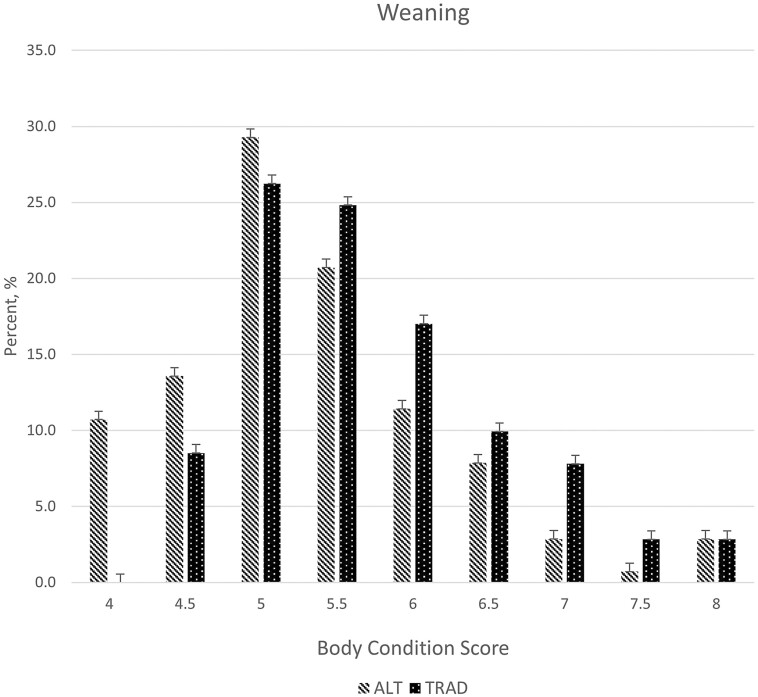
Body condition score at weaning for two cow-calf production systems. Treatments consisted of an alternative fall-calving cow-calf system utilizing partial-drylot, cover crop, and corn residue grazing (ALT) and a traditional, spring-calving cow-calf system utilizing perennial forages and corn residue grazing (TRAD). Body condition distribution was significantly (*P* < 0.01; SE = 0.55) different among the treatments. There was a shift in the ALT body condition distribution with a greater percentage of body condition scores of 4 and 5 compared to the TRAD treatment.

Breeding BCS distributions differed (*P* < 0.01) with a larger proportion of cows having a BCS of 5.0 and fewer cows with BCS scores of 6.5 to 7.0 for ALT compared to TRAD cows (Figure 7). Over 90% of cows in the ALT system had BCS between 5.0 and 6.0 at the time of breeding, suggesting that energy intakes were adequate for maintenance and lactation during the drylot period, which occurred directly prior to breeding. The greater BCS for the TRAD cows at the time of breeding suggests that their energy intake surpassed requirements for maintenance and lactation, and these cows were storing energy as fat. The timing of BCS measurements for breeding in the TRAD system occurs at peak biomass availability in smooth bromegrass paddocks (Figure 2). Although the difference in BCS between systems persisted through weaning (*P* < 0.01; Figure 8), the mean BCS for the TRAD system moved closer to 5.0 at weaning, whereas the ALT system maintained BCS from breeding to weaning. The cows in TRAD system were in a negative energy balance late in the grazing season and utilized stored energy for their requirements during lactation. The rotational grazing system used in the TRAD system appeared to be successful in maintaining diet quality, represented as diet IVOMD (Figure 3) and diet CP (Figure 4). This may suggest that cows had limited biomass late in the grazing season (Figure 3).

Although the mean BCS shifted lower for cows in the TRAD system, the range of BCS among cows in the herd appeared to remain consistent from breeding to weaning. The ALT cows appeared to have an increase in variability of BCS from breeding to weaning. As noted previously, over 90% of ALT cows had a BCS of 5.0 to 6.0 at breeding when they left the drylot. This proportion was reduced to 60% at weaning following grazing of oats, with greater proportions of cows being less than 5.0 and over 6.0. Although the mean BCS of ALT cows may have been similar from breeding to weaning, they became less uniform after grazing fall oats. This suggests that for some cows the oats cover crop was not meeting energy needs and for others it was exceeding energy needs during lactation.

Regardless of differences in BCS from breeding to weaning, the pregnancy rates were not different (*P* = 0.88) among treatments. A perceived concern with the ALT system has been an anticipation of reduced pregnancy rates due to high CP forage consumption. Lenz et al. (2019) reported CP of 18% in early November for late August and early September planted oats. In the current experiment, the CP of oats does not suggest that the forage was high in CP, and in fact, CP may become limiting late in the grazing period.

Calf performance is presented in Table 6. As designed, calf age at weaning was not different (*P* = 0.76) at 168 d for both treatments. Calf birth weight, not including the removed twin calf, did not differ (*P* = 0.35) among TRAD and ALT treatments. Previous research has also reported no difference in calf birth weight among forage and confinement-based systems (Perry et al., 1974; Anderson et al., 2013; Burson, 2017). Calf wean BW was 45 kg less (*P* < 0.01) for ALT calves compared to TRAD calves. As a result of lesser wean BW, kilogram of calf weaned per cow exposed was 49 kg less (*P* < 0.01) for ALT cows compared to TRAD cows. The reduced weaning BW in the ALT system may be related to the fact that summer-born calves have more environmental stress from birth to weaning than spring-born calves with much colder temperatures in November, December, and January (Table 4). However, similar responses to the current experiment have been reported in wean BW for forage-based cow-calf systems with calves being 18 and 17 kg heavier at the same age than calves from confinement cow-calf systems (Anderson et al., 2013; Burson, 2017, respectively). Alternatively, Perry et al. (1974) reported no difference in wean BW between confinement and forage-based cow-calf systems, whereas Neira (2019) reported greater wean BW for confinement raised calves compared to forage-raised calves. However, the authors attributed the lower wean BW for forage-raised calves to drought conditions on pasture. Additionally, Perry et al. (1974) reported lower ADG for February- and March-born calves from a confinement cow-calf system during the months of May and June, suggesting that those calves were not large enough to compete with their dams for access to the feed bunk. Differences in nutrient density of the diet, intake, calf access to feed, and weather conditions are likely major contributors to differences in calf weaning weights among different studies.

**Table 6.** Comparison of a traditional spring-calving pasture-based cow-calf system (TRAD) to an alternate summer-calving cow-calf system utilizing drylot and oats grazing (ALT) on calf performance

	Treatment^1^			
	ALT	TRAD	SEM	*P*-Value
Groups, *n*	8	8	—	—
Birth BW^2^, kg	39	40	0.7	0.18
Age at wean, d	168	168	1.1	0.76
Wean BW, kg	184	229	5.5	< 0.01
kg weaned/cow exposed^3^	150	199	7.2	< 0.01
Calf morbidity^4^, %	33.1	8.8	3.97	< 0.01
Calf mortality^5^, %	7.8	4.1	—	—

Treatments = alternative cow-calf system (ALT) calving in July to September and utilizing drylot, fall forage oat grazing, and corn residue grazing; traditional cow-calf system (TRAD) calving in April to June and utilizing perennial forage and corn residue grazing.

For twins, only the birth weight of the one calf selected randomly to remain in the experiment was included.

Kilogram of calf weaned divided by the number of cows exposed to bull.

Calculated by the total number of calves treated at least once from a group divided by the total number of calves in that respective group.

Calculated by the total number of calves that died from a group divided by the total number of calves in that respective group. Calves that died at birth or within the first 24 h of life were not included in mortality calculations.

It is possible that diet composition during the limit-fed, drylot period in the ALT treatment altered milk production. Milk composition and production were not measured in this experiment. However, in confined, limit-fed, lactating cows fed a diet with 55% distillers grains plus solubles and dietary fat up to 7.8%, milk composition or yield was not affected compared to a corn gluten feed diets with 3.0% to 4.3% dietary fat (Shike et al., 2009). Dietary levels of fat close to 5% from animal and vegetable sources fed to dairy cows lowered milk fat content but not milk yield (Coppock and Wilks, 1991). However, diets high in rumen undegradable protein, like in the current experiment, may repartition nutrients from milk production towards maternal body growth (Hunter and Magner, 1988; Wiley et al., 1991; Triplett et al., 1995).

An important consideration in the current experiment is that one calf from each set of twins was removed at birth, yet the ALT system generated significantly more twins. In the current study, when birth BW of only the singleton born calves from both treatments was analyzed, there were no differences (*P* = 0.28; 41 vs. 39 kg for TRAD vs. ALT, respectively; data not shown). Also, when the twins were removed from the analysis, weaning BW for TRAD calves (229 kg) did not change and ALT calves’ wean BW increased by only one kg (185 kg). This indicates that the weaning BW of the twins did not have a large impact on the overall growth performance in either system. However, over the course of two production cycles, the ALT system produced 11 more sets of twins than the TRAD system. Guerra-Martinze et al. (1990) estimated that twining increased the efficiency of beef production when twin calves were maintained in the system by 24%. Echterncamp and Gregory (2002) reported sets of twins had greater total birth BW (57.3%) and weaning BW (48.1%) compared to dams with single births. If these twin calves remained in the system in the current study, it might have increased weaning BW for the ALT treatment.

However, leaving twin calves with the cow has potential negative effects. Echterncamp and Gregory (2002) observed cows nursing twins had prolonged postpartum anestrous periods and a reduction in ovulation rate. In their experiment, cows nursing twins were sorted off and provided an additional 27 Mcal of ME/cow/d of a corn and corn-silage diet compared to cows nursing a single calf. Even though twinning cows were provided additional energy and were maintained in moderate body condition, there may be other factors impacting the fertility of cows after a twin birth. In the current experiment, cows that had given birth to twins in the first production cycle (*n* = 2 and 6 for TRAD and ALT cows, respectively) were all pregnant in the second production cycle. However, cows that had given birth to twins in the second production cycle (*n* = 3 and 10 for TRAD and ALT cows, respectively) had pregnancy rates of 50 (one TRAD cow removed from the experiment prior to breeding) and 80% for TRAD and ALT cows, respectively, after giving birth to twins. Other negative effects reported by Echterncamp and Gregory (2002) include a greater incidence of dystocia and lower survivability for twin calves.

In the current experiment, calf morbidity was greater (*P* < 0.01) for ALT calves compared to TRAD calves. Nearly one-third of the calves (33.1%) from the ALT treatment were treated at least once for morbidity purposes (i.e., bloat, bodily injury, coccidiosis, digestive issues, foot rot, navel infection, respiratory issues, and scours) compared to 8.8% of TRAD calves. In cycle one (2018), 27.8% of ALT calves were treated at least once compared to 10.7% for TRAD calves. In cycle two (2019), 38.5% of ALT calves were treated at least once compared to 7.0% of TRAD calves. Across both cycles, of calves treated for morbidity, the majority of treatments for ALT calves were due to navel infections (32.3%) and bovine respiratory disease (BRD; 27.4%). The majority of morbidity treatments for TRAD calves were due to BRD (20.0%), foot rot (20.0%), and clostridial infections (13.3%). Precipitation in 2018 before and during calving (July to September) for the ALT system was 145% of the 30-yr average for Lincoln, NE (NOAA, 2020). The objective for calving the ALT system from July to September was to target the time of year with drier conditions. This was not the case for 2018; wet pen conditions were likely the cause of the increased rate of navel infections in the ALT calves in that year (54.2% of treatments). In cycle two (2019), the majority of morbidity treatments for the ALT calves were BRD (36.8%) and navel infections (18.4%). These findings agree with Burson (2017), reporting greater probabilities of morbidity for confined-based calves compared to forage-based calves (64.5% vs. 2.5%, respectively). Warner et al. (2014) reported BRD occurrence in a fully confined cow-calf system for 2 yr at two separate locations. In year one, 26% and 0% of calves were treated for BRD from location one and two, respectively. In year two, 0% and 84% of calves were treated for BRD from location one and two, respectively. The authors attribute the differences among years and locations to variation in weather, stress, and exposure to other cattle. In the current experiment, of the calves treated for morbidity, BRD treatments varied for ALT calves among production cycles one (12.5%) and two (36.8%) with the cause of variation unknown but weather likely had an influence. Extensive or intensive production systems have inherent morbidity and mortality risks (Gunn et al., 2014). Smith (2013) reported that the greatest risks for survival of newborn calves include dystocia and difficulty at calving, weather and environmental conditions, and disease. Intensive cow-calf systems have increased animal to animal contact, creating a greater opportunity for pathogen transmission than extensive cow-calf systems. Other risk factors for disease transmission include cattle movement in and out of the operation, fence line exposure, and degree of confinement (Warner et al., 2014).

## IMPLICATIONS

This experiment evaluated the performance of a cow-calf production system utilizing drylot, oats cover crops, and corn residue compared to a traditional, forage-based cow-calf production system. It provides evidence that a partially confined cow-calf system does not negatively impact reproduction. There may be an increased probability of twin offspring for the alternative cow-calf system when breeding in the winter while grazing late-summer planted oats. Weaning weights may be substantially reduced in the summer-calving alternate system compared to spring-calving in a perennial forage-based system. However, alternative cow-calf systems, such as the one examined here, can provide options and insight for prospective or expanding cattle operations without access to perennial forage.
